# What does ‘good’ palliative care look like for children and young people? A qualitative study of parents’ experiences and perspectives

**DOI:** 10.1177/02692163231154300

**Published:** 2023-02-24

**Authors:** Diana Fields, Lorna Katherine Fraser, Jo Taylor, Julia Hackett

**Affiliations:** Martin House Research Centre, Department of Health Sciences, University of York, York, UK

**Keywords:** End-of-life care, palliative care, parents, qualitative research, child

## Abstract

**Background:**

Worldwide, around 21 million children would benefit from palliative care and over 7 million babies and children die each year. Whilst provision of paediatric palliative care is advancing, there major gaps between what should be done, and what is being done, in clinical practice. In 2017, the National Institute for Health and Care Excellence (NICE) introduced a quality standard, to standardise and improve children’s palliative care in England. However, there is little evidence about what good experiences of palliative care for children are, and how they relate to the quality standard for end-of-life care.

**Aim:**

This study explored how the NICE quality standard featured in parental experiences of palliative care for children to understand what ‘good’ palliative care is.

**Design:**

Qualitative study, employing in-depth, telephone and video-call, semi-structured interviews. Data were analysed using thematic analysis, informed by Appreciative Inquiry.

**Setting/participants:**

Participants were parents of children and young people (aged 0–17 years) in England, who were receiving palliative care, and parents whose child had died.

**Results:**

Fourteen mothers and three fathers were interviewed. Seven were bereaved. Parents were recruited via four children’s hospices, one hospital, and via social media. Good palliative care is co-led and co-planned with trusted professionals; is integrated, responsive and flexible; encompasses the whole family; and enables parents to not only care for, but also to parent their child to end of life.

**Conclusions:**

Findings have implications for informing evidence based practice and clinical guidelines, overall improving experiences of care.

**What is already known about the topic?**

- Worldwide, there are known inequalities in access to and provision of palliative care, and a major gap remains between quality standards, and what is being delivered, in clinical practice.
- There is a postcode lottery of accessing palliative care because of a fragmented healthcare system, with parents fighting the system to access palliative care and services.
- Terminology such as ‘palliative care’ is often misunderstood, has negative connotations and is often associated with imminent death.

**What this paper adds?**

- Misunderstandings around terminology such as ‘palliative care’ and misperceptions that the trajectories of children with life-limiting conditions are the same as adults, means children miss out on care that is highly valued by parents.
- There are still marked differences in parents’ experiences of accessing palliative care and end-of-life care services for their children, particularly for parents of older children and those with medical complexity.
- Good palliative care is care that is co-led and co-planned with trusted professionals working in palliative care; is integrated, responsive and flexible; encompasses the whole family, and enables parents to not only care for, but also to parent their child to end of life.

**Implications for practice, theory or policy**

- Accessing charity funded services does not fulfil the care gaps experienced by parents, indicating a pressing issue to meet the palliative care needs of children and young people.
- Parents ask for joined up thinking and continuity across hospital and hospice teams to provide a balance of intensive and palliative care support, which encompasses the whole family.
- There is a need for health professionals to undertake further training in palliative care and bereavement support, though further research is needed to understand professionals’ perspectives.

## Introduction

Worldwide, it is estimated that nearly 21 million children would benefit from palliative care input^1^ and over 7 million babies and children die each year.^2^ Prognoses vary markedly, with some children living only a few weeks or months and others living into adulthood despite families receiving a diagnosis in infanthood.^3^ In England, although many individual diagnoses are rare, as a group, children and young people with life-limiting conditions are a larger patient population than many other long-term conditions in children and young people.^4^

Palliative care, defined as ‘an active and total approach to care, which begins from diagnosis or recognition and continues throughout the child’s life and death’,^5^ is an important component of care these children and young people will require. It includes symptom and pain management, provision of short breaks, psychosocial and spiritual care, and end of life and bereavement care. In England, it is provided by a range of services, including hospital-based teams, which provide ongoing care for a child, for example: for children with cancer by a paediatric oncology team; or for infants born with a life-threatening condition, by a neonatal team. For other children, palliative care may be provided by a children’s community nursing team, specialist paediatric palliative team, or a children’s hospice.^6^ Provision also varies significantly by service, region and speciality.

Paediatric palliative care is not equally available in all countries, and whilst provision is advancing, a major gap remains between what should be done, and what is actually being done, in clinical practice. Internationally, various standards of care and guidelines have been developed to improve the quality of paediatric palliative care, for example: IMPaCCT (Standards for paediatric palliative care in Europe),^7^ the GO-PACCS project (Global Overview – PPC Standards)^8^; National Paediatric Palliative Care Clinical Guidelines, New Zealand; NHPCO Standards for Pediatric Palliative Care, USA; and in the UK, End of life care for infants, children and young people with life-limiting conditions: planning and management. However, there are known barriers to accessing palliative care,^9^ meaning families of children, even whom have similar healthcare needs, may receive a very different experience of palliative care.^8,10,11^ Existing standards are also not widely applied in clinical practice, therefore there is an urgent need for a critical revision and update of current recommendations and practices to promote a wider implementation of paediatric palliative care standards in all countries.^8^

In England, The National Institute for Health and Care Excellence (NICE) has quality standards, which set out priority areas for quality improvement, and are developed using a robust evidence base and a rigorous process. They are a set of specific, concise statements that act as markers of high-quality, cost-effective patient care, covering the treatment and prevention of different diseases and conditions; and highlight areas with identified variation in current practice. To help standardise and improve children’s palliative care in England, NICE developed a new clinical guideline in 2016^12^ and associated quality standard in 2017,^13^ although the guideline only applies to those aged 0–17 years. It sets out six quality statements about the care children with a life-limiting condition should receive (Table 1).

**Table 1.** NICE quality standards for end-of-life care for infants, children and young people.

Statement 1	Infants, children and young people with a life-limiting condition and their parents or carers are involved in developing an Advance Care Plan.
Statement 2	Infants, children and young people with a life-limiting condition have a named medical specialist who leads and coordinates their care.
Statement 3	Infants, children and young people with a life-limiting condition and their parents or carers are given information about emotional and psychological support, including how to access it.
Statement 4	Infants, children and young people with a life-limiting condition are cared for by a multidisciplinary team that includes members of the specialist paediatric palliative care team.
Statement 5	Parents or carers of infants, children and young people approaching the end of life are offered support for grief and loss when their child is nearing the end of their life and after their death.
Statement 6	Infants, children and young people approaching the end of life and being cared for at home have 24-h access to both children’s nursing care and advice from a consultant in paediatric palliative care.

Little is known about how the elements of care in the NICE quality standard feature in families’ experiences of palliative care, and what, for families, good palliative care is. At this time, we do not know whether good palliative care could differ from, or expand upon, the current NICE quality standard. Existing studies provide important insights about certain elements of palliative care, for example decision-making, Advance Care Planning; or highlight points in the care pathway that could be improved, such as around diagnosis, end of life, or when to introduce palliative care. However, they tend to provide fewer insights about provision and how this is experienced.

To our knowledge, there are no robust or recent UK studies exploring how the NICE quality standard features in parental experiences of palliative care for children or sought to understand what good palliative care looks like. This study aims to address this evidence gap and hopes to inform future service developments and clinical guidelines for children’s palliative care.

## Methods

This study involved thematic analysis of in-depth interviews with parents, informed by Appreciative Inquiry.^14^ It focussed on drawing out what works well (Discovery phase) and what could be (Dream phase). Appreciative Inquiry has been successfully used in a UK study looking at needs of children with a life-limiting condition in one region of England,^15^ and has also underpinned qualitative research exploring other areas of health.^16–19^ There are five foundational principles (Table 2), which focus on what works well and draw out positive experiences and imaginings from participants and the research process itself.^14^

**Table 2.** The foundational principles of Appreciative Inquiry.^14^

Principle	Definition
The Constructionist Principle	Reality is socially constructed through language
The Simultaneity Principle	Change begins from the moment a question is asked
The Poetic Principle	Our choice of what we study determines what we discover
The Anticipatory Principle	Our image of the future shapes the present
The Positive Principle	Positive questioning leads to positive change

### Population

Eligibility criteria were: parents or legal guardians of children and young people (aged 0–17 years) who were receiving palliative care; or whose child had died (aged 0–17 years, between 3 months and 3 years ago); where palliative care had been discussed (i.e. their child was supported by a palliative care service or had an Advance Care Plan or their child had died).

### Sampling

We recruited from organisations purposively across England to ensure variations in care provision, and included areas diverse in terms of their geography, ethnicity and socio-economic status. Initially, all eligible parents were invited to participate. Subsequently, a purposive sampling strategy was used to ensure that the sample included similar sub-groups of parents whose child was currently receiving palliative care and parents whose child had died, and reflects the diversity within this population of parents both in terms of their own characteristics but also in terms of their child’s condition. These purposive sampling criteria include type of diagnoses in the child, duration of illness and prognosis, and age and capacity of child, all of which have been identified as potentially affecting access to palliative care.

### Recruitment

Charity organisations’ social media, four children’s hospices and 1 hospital supported recruitment. Health professionals discussed the study with eligible parents either face-to-face or by telephone. All those interested were given a study information pack in person, or by post/email. Packs contained an invitation letter, parent information sheet, a consent-to-contact form, and a pre-paid return envelope addressed to the study team, marked confidential. Parents could also return the consent-to-contact by email. Separate study documents were used to recruit bereaved parents and parents of a child with a life-limiting condition. Parents who were separated or divorced both received an invitation pack for the study. For all families, parents were offered the choice of being interviewed together or separately if more than one parent of a child wished to take part.

Parent advisors were keen for us to recruit via parent facing organisations to avoid healthcare practitioner gate-keeping.^20^ We advertised via charity organisations’ social media pages, for example: Facebook and Twitter. Parents who were interested contacted the study team either by telephone or email and sent a study pack, as above.

The study team contacted all parents who completed a consent-to-contact form, to discuss the study, answer questions, check eligibility, and provide a consent form. Once consent was completed, arrangements for an interview were made. Parents were recruited October 2021–March 2022.

### Data collection

In-depth semi-structured interviews explored parents’ accounts of palliative care for their child and wider family support, introduction of palliative care, discussions and planning, what aspects of care enhanced quality of life, and unmet needs. For bereaved parents, care provided during end of life and bereavement was also explored. Prompts for the NICE quality standards were used to ensure these were covered across the topics. The semi-structured nature of interviews allowed for collection of rich data, comparison between individuals, and exploration of similarities and differences based on sampling characteristics.^21^

Parents could choose a video call or telephone interview (data collection period during COVID-19 restrictions). Where both parents wished to participate, individual or joint interviews were offered. Interviews were conducted by the first author (DF; a female, applied health researcher, parent, previously unknown to participants).

Field notes recorded key points, observations and thoughts during and after interviews. During analysis this aided interpretation and encouraged researcher reflexivity.^22^ Interviews were audio-recorded and transcribed verbatim. Interviews continued until information power had been reached.^23^ The concept of information power is based on five dimensions, which have an impact on the power of the study: study aim, sample specificity, use of established theory, quality of dialogue, and the analysis strategy.^24^ Throughout data collection, we concurrently assessed our study against these five dimensions to decide when information power was achieved. We posit that we have a narrow study aim, participants who hold characteristics that were highly specific for the study aim, theoretical underpinning, strong interview dialogue, and an in-depth analysis. Therefore, with these factors, we achieved information power with our sample size.

### Data analysis

Interview data were analysed using thematic analysis^25^ and informed by Appreciative Inquiry principles,^14^ specifically data collection and analytical processes, with steps repeated when necessary.^25^ We shaped the questions we asked parents, ensuring a focus on positive experiences and imaginings, as well as negative experiences parents wished to share.

First, DF listened to all audio-recordings. Interview transcripts were read and re-read for familiarisation, with notes taken on key concepts, generating ideas, issues and experiences. Data were systematically coded inductively and deductively, across all transcripts, identifying all data in relation to each code. A priori codes pertaining to the NICE Quality Standards were used to ensure these aspects of care were explored explicitly. Codes were grouped into descriptive categories to explore means of codes and potential relationships between codes. Analytical themes were developed by summarising and seeking to understand coded data and descriptive categories and further explore relationships.^25^ NVivo 12 was used for managing data.^26^ D.F. worked reflexively with J.H. to review coding, discuss themes and aid theme development.

### Ethical considerations

West Midlands – Coventry and Warwickshire Research Ethics Committee approved the study (REC reference: 21/WM/0152). Due to the sensitive subject matter of this study, there were various ethical considerations to consider, both for the participants and the researchers themselves.

Parents need to be able to make informed decisions about participating. Talking about their child’s palliative care, and for bereaved parents the death of their child, may cause some parents distress. In order to minimise this, this and the topics that would be expected to be covered in the interview, were explicitly acknowledged in the participant information sheet and repeated in telephone conversations with interested parents. All communications made it clear that parents could ask to have a break, stop the interview at any time, or re-schedule it for reason, or ask not to answer a specific question or speak about a particular issue. Parents were reminded about this at the start of the interview.

The possibility of becoming upset due to the topics discussed was explicitly addressed by the researcher at the start of the interview in order to put parents at ease and also to impart to them a sense of control over the interview process. During the interview, the researcher was attuned to notice signs of unease or distress. Towards the end of the interview, the topics covered served to lighten the interview. At the end of the interview, the researcher asked parents whether they would like to receive a follow-up call, after the interview, at a time chosen by the parent. This was used: (a) to check the parent was happy for their interview to be used for the study; (b) as an opportunity for the parent to raise anything they reflected upon since the interview; and (c) to signpost them to organisations for support if required.

In addition, researcher well-being was an important ethical issue to consider and was addressed throughout the study. The research team have extensive experience of working on studies, which present similar challenges and have a high emotional load. In preparing for data collection, two pilot interviews were conducted with members of the studies Patient and Public Involvement panel and then discussed in terms of questions and topics asked, flow of the interview, emotional load and well-being. There was a pre-determined escalation procedure in place and the Principal Investigator was on call during all interviews to provide support if required. The researcher and the Principal Investigator then debriefed after every interview and had regular supervision meetings throughout the study, particularly during data collection and analysis, which are times when the emotional load of the study were greater.

### Patient and public involvement

Parents reviewed the study proposal and actively contributed to the study design. They highlighted the importance of: including parents of children with a life-limiting condition and bereaved parents; diverse end-of-life care experiences; developing a sampling strategy; and advertising through social media. Parents helped prepare participant facing documentation and piloted and refined interview topic guides. They provided feedback on initial analytical themes, providing new avenues of thoughts, and clarified aspects of the data.

## Results

### Sample

Twenty parents returned consent-to-contact forms and met inclusion criteria. However, three parents chose not to continue. Therefore, 14 mothers and three fathers were recruited, representing 16 families, each with one child who had received, or was receiving, palliative care. One was a joint interview with a mother and father. The remainder were individual interviews (mothers: *n* = 13; fathers: *n* = 2). Seven were bereaved parents, bereaved between 12 and 24 months (median = 19 months) (Table 3). Nine video call and seven telephone interviews were conducted. Mean interview length was 77 min (range: 35–180 min).

**Table 3.** Characteristics of sample.

*Bereaved parents children’s characteristics*	
Parental awareness of life-limiting condition	
Before birth	1
From birth	5
Child gender	
Female	5
Male	1
Place of child’s death	
Home	1
Hospital	1
Hospice	4
Received hospice care	
Yes	5
No	
Occasionally	1
Child had other siblings	
Yes	4
No	2
Advance Care Plan	
Yes	6
No	
Verbal/non-verbal communication	
Verbal	0
Non-verbal	6
Age range of children at time of death	7 weeks to 17 years
Condition type of child	
Neurological	2
Metabolic	2
Congenital	1
Congenital/neurological	1
*Parents of a child with a life-limiting condition*	
Child gender	
Female	4
Male	6
Parental awareness of life-limiting condition	
Before birth	1
From birth	8
Other	1
Receiving hospice care	
Yes	8
No	2
Siblings	
Yes	8
No	2
Advanced care plan	
Yes	9
No	1
Verbal/non-verbal communication	
Verbal	1
Non-verbal	8
Speech has changed to non-verbal	
Age range of children at time of interview	4 years to 17 years
Condition type of child	
Neurological	2
Metabolic	3
Congenital	3
Congenital/neurological	2

The NICE statements featured heavily in the themes and sub-themes (Table 4). Table 5 summarises the Dream Phase^14^ wishes of parents and their continuing needs.

**Table 4.** NICE quality standards for end-of-life care for infants, children and young people and derived themes and subthemes.

Statement	Description	Theme	Subthemes
Statement 1	Infants, children and young people with a life-limiting condition and their parents or carers are involved in developing an Advance Care Plan.	Introducing Advance Care Plan Development and reviewing of Advance Care Plan and palliative care needs	Communication, and timely discussions No key person in charge Reviewing plans is a tick box exercise The need to advocate for your child and to drive care Positive feelings about Advance Care Plans
Statement 2	Infants, children and young people with a life-limiting condition have a named medical specialist who leads and coordinates their care	Lack of a coordinator	
Statement 3	Infants, children and young people with a life-limiting condition and their parents or carers are given information about emotional and psychological support, including how to access it	Mixed experiences about being informed of emotional and psychological support	
Statement 4	Infants, children and young people with a life-limiting condition are cared for by a multidisciplinary team that includes members of the specialist paediatric palliative care team.	Feelings of luck and guilt accessing care and resources from charities Misconnect between services, professionals and parents Treated like you matter	
Statement 5	Parents or carers of infants, children and young people approaching the end of life are offered support for grief and loss when their child is nearing the end of their life and after their death.	Experiences of grief and loss support	
Statement 6	Infants, children and young people approaching the end of life and being cared for at home have 24-h access to both children’s nursing care and advice from a consultant in paediatric palliative care.	Facilitating choices at end of life	

**Table 5.** Parental wishes and unmet needs.

NICE statement	Parental unmet needs	Parental wishes	Exemplar quote
Statement 1 Infants, children and young people with a life-limiting condition and their parents or carers are involved in developing an Advance Care Plan.	Implications of not having a definitive timescale for life limiting condition – lack of finances Implications of not having a definitive timescale for life limiting condition – mires access to support and care	Professionals introduce palliative care in a positive way Building of relationships and trust Educate society what palliative and hospice care really means Joined up thinking and consistency of palliative care and support across teams	It is about the end but it’s about so much more than that, it’s about living. It’s not, well, there’s nothing else that can be done. It is definitely about living as well and making the most of whatever time we’ve got. (0211) It’s very painful to have to keep going through something that clearly is written down, we’re not idiots and we’re not in denial. . . You know it and you do talk about it when it’s appropriate and you talk about the counselling, but it’s like scratching a wound. You keep, you know, lifting the scab off that wound. What’s the purpose here for that? (0414) Treating the family as a whole, and not treating a disabled child as an appendage to the family. (0601) I think a lot of it comes down to palliative care being part of everybody’s remit. I’m sure doctors must have some training on it, I have no idea how much, because death is going to be part of doctoring, isn’t it? So there must be something on it. But I think there needs to be much more of a whole team approach, that everybody is aware of it, knowing that palliative care is very much a living thing, it’s not the end. It’s a way of living with your condition and making the most of life, not just an opt out. They could have been a lot better. (0211) So communication, joint working, I think those are really important and they would make a big difference to a lot of people. (0211) I think living this life is incredibly difficult for people. It’s not just having a child that will go on into adulthood. We’re living, we’re nursing, we’re doing everything, and we don’t have the equipment, the facilities, to be able to do it properly, as many families I think have, you know. . ..I don’t have enough resources. I don’t have enough money. I don’t have enough people power. I don’t have enough space. We don’t have what we should have to make that function. We are a far cheaper option. She can go spend 52 weeks a year at school, it’s £3000, £4000, £5000 a week for her. If we were just given a smidgeon of that to have an extra room on our house, or to have somebody to take [name of child] swimming or out to climb trees or – you know, it would help enormously. (0604). One of the things we have come across in the past as well is charities like [name of charity], when you start filling in the forms, it is not quite as blunt as, ‘How long have they got left?’ but we had trouble accessing that because we haven’t got a timescale. You know, to say they could live until they are 40, we are then not priority. If you have been given a diagnosis for your child and you have got 12 months, you go out and you start doing Disneyland and meeting the stars and whatever. But when you have got an open-ended timeframe, it wouldn’t be right to live like that and keep going, ‘Well, [name of child] might die in a years’ time so we want to see [name of singer] or whatever’. We can’t live like that, we have to live as if it is years in the future. (0619)
Statement 2 Infants, children and young people with a life-limiting condition have a named medical specialist who leads and coordinates their care	Lack of a coordinator/joined up working		So, communication, joint working, I think those are really important and they would make a big difference to a lot of people. (0211)
Statement 3 Infants, children and young people with a life-limiting condition and their parents or carers are given information about emotional and psychological support, including how to access it		Further Support – pre-bereavement, where parents can see for themselves what is on offer	I think there is an awful lot of bereavement support, but there is no pre-bereavement. There is nothing before. We know where they have their bedrooms, we know that there is a set of double doors, and behind the double doors there are these little bedrooms for children that have passed away where they lay them in the freezer, in the cold rooms, whatever. We have never seen them, and we think that is something that should be offered, or parents known about. I would love to go and see one, so that on the particular day it is not a shock to walk into this room and see. I would like to know in advance, and I think that is something that should be offered by hospices. Again, on an individual basis, not every parent is going to want to be reminded of that, they are not going to want to go and see it because it is too painful. But for some parents, let us go and see what it looks like. (0619)
Statement 4 Infants, children and young people with a life-limiting condition are cared for by a multidisciplinary team that includes members of the specialist paediatric palliative care team.	Lack of space and equipment Treatment, symptom management and pain relief	Development of systems of coordination – access to palliative care support Development of systems of coordination – online medication system	So, we have no lounge. [Name of child] lives in the lounge. That’s where her bed is. So, we’re in quite cramped conditions, plus all of [name of child]’s equipment, which keeps growing. Storage isn’t taken into consideration through the DFG for people. That’s really difficult for people. It’s difficult for us. There is no space for everything. [Name of child] has a ventilator. She’s got two suction machines, a nebuliser, feeding pump, oxygen, and that’s in her room. We’ve got a tiny sofa-bed in her room where we don’t function as a normal family anymore. I long to sit and watch telly on a sofa. I want to watch telly with a cup of coffee on a coffee table. We don’t eat in the room because it’s her bedroom, but it’s also our lounge. We don’t have that space. And I think environment plays a huge factor in people’s lives. (0604) So when we went into the final days, there was like two transitions between daylight and night, and day, and I think during that time the administration of pain management drugs, it was slower than what it should have been and I think there was a point where [name of child] suffered and had more pain that what he should have because there were, the hospice and the sort of team around him during that sort of night period or very early morning, they were too slow to increase the dosage. . . So I think that that could have been better. (0103) A national database of children, where we could tap into nursing advice if we needed it, they’ve got all of [name of child]’s records, so should we need hospital care while we’re away. I just think it would make families’ lives so much easier, and improve that. (0604) If they can get the online thing working they’ll be able to just print off a copy. . . Their idea is before they visit us they’ll just print off a copy of the up to date MAR and bring it out and then they’ll be able to give everything. So we’re looking forward to that day. (0211)
Statement 5 Parents or carers of infants, children and young people approaching the end of life are offered support for grief and loss when their child is nearing the end of their life and after their death		Further psychological support at diagnosis – better signposting and how diagnosis impacts life	Whether that is the paediatrician, the oncologist or whoever it is, it’s an enormously difficult task I think, is reading you as individuals and your child of how they engage in that conversation and get it right. Because it must be an absolutely minefield and they’re not always going to get it right. That probably isn’t always through their lack of trying, they will have tried their hardest. I mean it must be one of the hardest conversations to have. Because it is very difficult to know and gauge the right time to have a conversation and a family might not be ready but it may still be the right time. Because particularly with some palliative care, it’s end of life, you don’t have the luxury of time. (0414) When we first met with the specialist and he just went, ‘Yeah, she could have an end of life condition’. At that point we would have expected some signposting. You know, we fully understand there is no timescale, but even back then he could have been signposting us to other avenues of where to look and what we might need to look at in the future. (0619) What does that mean being tube-fed? I don’t understand actually because I’ve just realised I’ve never met anybody before who can’t eat.” In fact it wasn’t even something I had ever really considered, I don’t think it had really ever occurred to me how they ate, it’s such a fundamental thing that you just assume everybody does it. And now it feels really stupid and maybe I did know that they were fed by a tube, but why would I have known? (0618)
Statement 6 Infants, children and young people approaching the end of life and being cared for at home have 24-h access to both children’s nursing care and advice from a consultant in paediatric palliative care.		Further financial support – reduce inequalities	There really needs to be a formal palliative care team set-up for every hospital, which provides support 24/7, if we could get him home, he could have come home. I think it needs to be offered to all children with life limiting conditions because it’s not fair that your child is dying but not of cancer, so they get the hospital, but this child’s dying of cancer, they can be at home in their own bed with their family around them, that’s not fair. (0103)

**NICE Statement 1** – Infants, children and young people with a life-limiting condition and their parents or carers are involved in developing an Advance Care Plan

### Introducing an Advance Care Plan

Parents often described the introduction of an Advance Care Plan as a difficult and emotionally laden conversation that some found difficult to process. This was sometimes compounded by a lack of clear communication from professionals about planning one, with some parents experiencing professionals avoiding these difficult conversations.

I think we were told on a Friday or Saturday that [clinician] was coming in early next week and we were like well, if there’s something to say just tell us now but you could certainly tell that no one else wanted to be the bearer of bad news. (0103)

Initial discussions about Advance Care Plans mostly happened around the time of referral for palliative care. Experiences varied however, with some describing a single conversation and others a more gradual process. Instances where conversations went well were when professionals were optimistic and focused on improving quality of life, rather than just focussing on end of life. Positive discussions about palliative care were also based on professionals giving parents the time they needed, which varied across families, to accept their child’s diagnosis and the need for palliative care. Ensuring palliative care was communicated as a life-long intervention also helped to ensure the focus was not just about end of life.

I didn’t really understand, I wouldn’t even put the referral through or get in contact with our local hospice . . . So the nurse at the time, she was absolutely brilliant, she did it all for me. Our local hospice got in contact with me and again I was like, “I don’t want to do this, this is too real”, so my emotional feelings were really probably quite negative ones because I was like, “No, I don’t want to be here. I don’t want to be doing this. (0417)

She doesn’t [have an Advance Care Plan], but that is on our list of things to do, we’ve talked about looking at it, but it’s been difficult because it’s something that I want to do but her mum won’t want to do it. She doesn’t feel comfortable or likes the idea because it seems, kind of, final, and it’s not something she wants to think about. It was a very abstract idea, and at the time it didn’t really bear any relation to the reality of our daughter being that ill. (0601)

### Developing and reviewing of Advance Care Plan and palliative care needs

#### No key person in charge

Parents reported numerous professionals and services being involved in their child’s care and welcomed their involvement in planning. However, there would often be *no key person in charge*, driving care, leaving parents feeling frustrated, stressed, and burdened with care coordination for their child.

What we really lack is a coordinator for [name of child]’s care, for somebody, that central person, which – when [name of child] was taken on by the palliative care team, that was what they were sold to us, that, “This is your key person, they will liaise with the right person, give advice. (0604)

### Reviewing plans is a tick box exercise

After an ‘initial flurry of lots and lots of stuff’ (0618) when setting up an Advance Care Plan, parents described feeling somewhat abandoned and responsible for securing their child’s care and support, especially as plans were not updated or reviewed regularly.

We had all these people around us telling us all this stuff but then everything just kind of disappeared, it felt like it was almost as quickly as it came. It was like: you’ve got your chair, you’ve got your printout with your exercises on it, fill your boots, you’re done, that’s it – go live your normal life. (0618)

Where plans were updated, parents explained this played little part in their child’s care.

People come round every now and again to update her care plan, but we don’t see a lot else. (0604)

#### The need to advocate for your child and to drive care

Parents described mixed experiences of driving care. Parents of older children, children with medical complexity, or those with increasing or changing needs, often described the ongoing fight to get and maintain care. They highlighted that when navigating palliative care services, much of their time would be spent sourcing services that were not in existence.

I do believe that junior members of staff haven’t got a blooming clue what it is. They’re petrified to follow it. But they need to look at the care plan and follow that through. . .it’s not a known condition and a well-trodden path, it’s very scary for all concerned and somebody senior needs to take control. It’s not NICE Clinical Guidelines that tell you what to do, it is experience that tells you what to do and yes, the guidelines aren’t even in place for these complex children. (0606)

However, the parents of babies described a very different perspective of palliative care, where they felt ‘very well supported and informed’ (0308). Factual information was readily provided, enabling parents to make difficult decisions, without having to fight and advocate need.

If we wanted information we could get it, but the decision, everyone was quite clear in saying that there’s no right or wrong choice because it would be up to us to make that decision. So everyone was quite clear on, here’s the facts, and it’s effectively up to you. (0103)

### Positive feelings about Advance Care Plans

Parents explained that having a plan took away fear of the unknown and brought a semblance of calm to a very difficult, sometimes frantic, situation.

We welcome anything that is trying to plan round something that you can’t control. . .. and his needs and his future is something we have no control over whatsoever. Whatever will happen will happen. All that we can do in that is try and plan as best we can for what we face or for the situation we are dealing with. So having a plan in place that means that you have some semblance of control in a way. It’s always where you don’t want to be and you always worry about it because you don’t know what the outcome is going to be. . .But having a plan means that you know at least people will do the right thing and it can happen quickly. You don’t have to explain everything. It’s I suppose reassuring. (0414)

For some parents, talking about and processing the implications of having an Advance Care Plan and what this meant, was difficult, but they acknowledged it was an important step to take.

I think I noticed right well we’re in hospital a lot more, options are starting to run out and I was just having lots of thoughts. . .. She was just getting frailer, and I just thought if we were to ever be in a situation where she did need to be resuscitated or something I just thought there is just no way this tiny, tiny body is going to cope with that, and I wouldn’t want to put her through more unnecessarily, I didn’t think that that would be very fair to her. So, we spoke about it with her palliative care doctor. (0020)

Parents felt reassured that in planning an Advance Care Plan, this reduced worries about being blamed for doing something wrong, and would help mitigate any conflicts between themselves and professionals, particularly at end of life. Having a plan meant difficult, upsetting decisions about what to do had already been thought about and agreed.

However heart-breaking that would be, it would be agreed and there’d be no one blaming you. (0510)

But having a plan where another medical person has said, you do this, takes us out of having to have that fight. Which is really reassuring because you don’t want to be fighting with somebody over. . .you need to give the drug whether you agree with it or not. (0414)

Parents perceived successful planning as key, enabling their child to be as independent as possible, where needs were met ‘no matter how modest they are’ (0601) and where all the family’s needs were considered, providing ‘very holistic care’ (0308). Of key importance to parents, were plans for end of life, which considered the family as a whole.

It’s for us as a family unit, not just this is what [name of child] needs, but this is what the family needs, and the family’s needs will help [our child] achieve her needs by providing her a calmer, happier environment without additional stresses and worries of everything else. (0601)

**NICE statement 2** – Infants, children and young people with a life-limiting condition have a named medical specialist who leads and coordinates their care

### Lack of a coordinator

Parents talked about many different professionals’ involvement in planning/coordinating care, but having no one person or service co-ordinating or driving care. In many cases they had to drive this themselves, finding it stressful, time-consuming, challenging and uncertain.

My son would not have lived as long as he did without us taking some responsibility, which I think is quite typical of a lot of families. There’s a lot of families where that is not something they can do but I think there is a lot of families that will do that and yes, we effectively case managed our child. (0606)

Having professionals who supported and empowered parents to make decisions about their child’s care was highly valued, and eased the burden of acting as care co-ordinator for their child.

We found that the paediatrician was extremely good at explaining the benefits and the drawbacks of certain offerings, certain interventions. He explained to us in a way we actually understood. We found him to be really excellent in making clear what our decisions would mean. We always felt that we were the ones in charge in making those decisions. We never felt pushed. . . Everyone was always really respectful of our wishes. (0308)

Being able to contribute to multiagency meetings was described as the main way to sort out uncertainty in the absence of a named co-ordinator, and provided them with ‘more of a concrete plan about what things are available and what next steps to take’ (0601).

**NICE statement 3** – Infants, children and young people with a life-limiting condition and their parents or carers are given information about emotional and psychological support, including how to access it

### Mixed experiences about being informed of emotional and psychological support

Some parents described how they had needed psychological support after their child’s diagnosis, but had not been offered this.

For it to be more human as well, because the doctors are sometimes a matter of fact, and sometimes very clinical. The offer of, “do you need support afterwards”, as in parents, after the conversation say “do you need to talk somebody”, because sometimes that gets overlooked, sometimes parents end up with a bombshell in their lap, their child is possibly going to die, and they just get left to deal with it. (0601)

Where parents were offered support and were able to have difficult conversations about end of life, they were able to develop their confidence, empowering them to look after their child and their needs.

I suppose they’re helpful with my emotions as well as helping me to understand that I’m not useless, and it’s not just that they’re there, that they can do things, that I’m capable and I can do it too type thing. They’ve helped me a lot with knowing that I can help him as well. (0415)

Parents who were engaged with a hospice, described how they were supported emotionally and psychologically, and encouraged by hospice staff to make positive memories before and after their child’s death, which they valued. They also took comfort from knowing they would always be part of the ‘hospice family’.

So [name of hospice] said to us from the very beginning that their support would involve from [name of child] life to her death. And it would never expire, that support. So you know, being three at the time, if he [sibling] was ten and wanted to speak to someone, they would offer that. It would always be available, we would always be a family. (0308)

However, the pandemic, for some parents, was reported as a limitation to them accessing emotional and psychological support.

Everyone’s now only started to open their services up, so no, I haven’t [accessed emotional support]. I haven’t at all. But like I said, there is groups, like [anonymised named group], there’s a parent forum, which is nice because people can discuss their feelings and their emotions. . . but nothing, no groups to actually go to. (0616)

**NICE statement 4** – Infants, children and young people with a life-limiting condition are cared for by a multidisciplinary team that includes members of the specialist paediatric palliative care team

### Misconnect between services, professionals and parents

Parents were drawn from a wide geographical area within England. Therefore, perspectives highlighted the misconnect between services in areas, and between areas of the country, professionals and parents.

Parents described going through constant rounds of assessments to access different elements of care provision and support. They highlighted a lack of coordination and sharing of information between services and as a result having to act as the lynch pin, which was frustrating and time consuming.

When [name of child] was taken on by the [name of palliative care team], that was what they were sold to us, that, “This is your key person, they will liaise with the right person, give advice.”. . . I’d CCed [name of child]’s neurologist into it [an email]. He basically asked, “Has anyone come back to you?” And I was like, “No”. And then the palliative care team gave me a mobile number that I could call. And I was a bit like, “Oh okay, I’ll just call them”. But I guess what I would expect from it was, “Yes, I’ll liaise with this person, I’ll liaise with that person and coordinate that”, so I’m not chasing around all the different teams [child’s name] is under, the [names of 5 teams] and possibly a few others, that that pressure would be taken away. Because there’s a lot of chasing and coordinating that I have to do, whereas I assumed that was the role of the palliative care team. (0604)

For parents, this misconnect between services resulted in a lack of consistency, in and between areas. They recognised parents in other areas may not be supported by a team of multidisciplinary professionals, or have access to the same services.

I suppose maybe the hard thing is there are families out there that don’t get the referral through to the hospice and if we didn’t have the hospice I’m really not sure what we would do, which is obviously depressing to think about. And very depressing to think that there’s no government funding for them. That they provide an enormous service that is so exceptionally valuable for people in a very difficult position. That’s done entirely out of funding that they have to generate themselves. And, whilst we are lucky that our hospice is able to provide those services, if we lived in another area, potentially we wouldn’t get those services. (0414)

In practical terms, the misconnect between different services, and lengthy waits commissioning resources, meant a lack of urgency, which parents perceived as detrimental to their child’s wellbeing.

 “This is why it needs to be done so urgently because otherwise she’s going to be stuck at home until she gets a wheelchair”, to me that was the most important thing, except for the fact that whilst she didn’t have a wheelchair, not only was she going to be stuck at home, she was going to be stuck in bed because we had no equipment at home that was suitable for her. (0618)

Conversely, parents who reported co-ordination between services and teams working together, described good palliative care support and a package of care that met their child’s needs from diagnosis until end of life.

We all sit around the table, whoever needs to be there at the time, and that has been really helpful, because a lot of the time, not so much through health, but certainly through local authority type stuff, it is always a fight, it is always a battle. . . But when you have a whole group of professionals sitting around a table, if five or six professionals say they have got to have one, that single individual professional sort of feels accountable and thinks, “Well, if everybody is going to sort of gang up on me, I better sort it”. So, they are helpful in some respect. But it also keeps everybody on the same page. (0619)

#### Feelings of luck and guilt accessing support from charities

Parents experienced feelings of luck and guilt, about their reliance on charitable funding to access specialist palliative care support and vital equipment for their children.

I had a bit of a chat with [name of hospice], and then that’s when I found out that [child’s name] hours came out of their charity funding and not commissioned funding. So, I can’t say, “Oh well, I want more charity from you”. I just can’t say – it’s not a need. There are people at far greater need than we have. But I have gone back to children’s services to say, “There are huge gaps in the day with . . . that are missing and really should be funded”. (0604)

Some families factored this into their decision-making – whether their needs should be prioritised over other children when resources were finite and support provided by a charity.

It was like I didn’t want to take from a charity whose actual focus was people who were properly on the breadline, whereas this felt like a slightly different and easier to justify to myself that I wasn’t being selfish by asking them if they would buy her this chair – I can’t remember what it’s called now, but honestly it was the best piece of equipment that we ever got. . . It cost just over one thousand pounds, we would never have been able to buy it ourselves. (0618).

#### Treated like you matter

Parents valued support they received from palliative care specialists and staff who worked in palliative care services, describing their skills in having difficult conversations, treating their child like a person with respect and compassion. They also valued staff who provided their families with empathy, acknowledgement, familiarity, stability and sense of belonging,

I think that the hospice are fantastic. They are very good at saying all the services that they provide and checking in with you. These are people who are regularly dealing with end of life and yet they can remain positive. And that is astonishing. But it does make a massive difference to how we feel about it. Because somebody smiling at you, somebody cracking a joke, just the world goes on. . .they do acknowledge what’s going on. But they work to make the best of what you have. (0414)

They treated her like a person, a little girl. She was a little girl and they could see the person inside the body. (0602)

Parents described how professionals who did not work routinely with children requiring palliative care, were less likely to show the same compassion and understanding. Some parents believed there was a lack of training, skills and confidence among these professionals.

Just because she’s palliative, she’s still alive and needs the care. Sometimes I feel a little bit that those services, they’re not trying to make you feel dumped but they’re because they sort of hear palliative, oh well, there’s probably not much we can do. I know it’s logical but it can feel as if everything’s ending for your child almost before she’s died, she is still here, it’s caused a few tears. (0510)

**NICE statement 5** – Parents or carers of infants, children and young people approaching the end of life are offered support for grief and loss when their child is nearing the end of their life and after their death

### Experiences of grief and loss support

Parents reported mixed experiences of sourcing bereavement support and differing opinions among professionals about when it should be offered. Some were told it was too early for bereavement support, whereas others were asked if they still needed it after a year.

I asked for bereavement support and I was told that it was too early. . .And then I eventually got in contact with the GP who put a referral through to the local hospital and my first telephone conversation with them was, “Well, do you really still need it?” because it was a year since she died. (0602)

Parents who reported good bereavement care described early support that enabled them to make lasting memories of their child, both in the hospice or hospital, as well as psychological support that included the whole family. Parents were particularly appreciative of the ‘many little touches’ (0020) which helped to make positive and precious memories out of their situation, before and after their child had died.

I think on the first or second day we were there we did footprints with [name of child. They took the little clay moulds of her hands and her feet which we have got which are just so beautiful. They helped us take photos of her first bath. And we have access to the memorial garden which we visited on her first birthday. We went there which was lovely to be part of that. (0308)

**NICE statement 6** – Infants, children and young people approaching the end of life and being cared for at home have 24-h access to both children’s nursing care and advice from a consultant in paediatric palliative care

### Facilitating choices at end of life

Some parents described negative experiences of accessing 24-h care at home during end of life and the limited amount of time 24-h end-of-life care was offered to them. This resulted in periods of intensity of end-of-life care at home and was perceived as reducing the quality of time parents had left with their children. At the end of life, parents wished for professionals to work on ways of reducing the responsibility on them for their child’s care needs, enabling them to remain as parents and move away from their role as carers.

I couldn’t spend quality time with her during the day. Because her care was so intense it was basically ITU care at home. . .There [at the hospice] I was able to have quality time with [name of child]. And I could join in with all the activities as well. It was like being a mum, instead of being a carer. (0602)

For all, having choices over preferred place of care was important and decisions would be based on what each environment could offer the whole family. Some parents believed they would feel more supported with professionals around them, particularly when their child was at the end of life. They took comfort in knowing they could access the support of professionals yet in a homely environment. This choice allowed them to separate memories and home life after their child had died.

So it separates that event from being in the family home (0601)

I got comfort in that and the fact that people were there 24/7 to look after [name of child] and to provide the medication because it was when [clinician] said that they don’t provide 24 hour round the clock care. So basically they’re just 9:00 to 5:00, so if we got [name of child] home then we would have to do the care plan and the medications and I couldn’t live with myself if I gave him the last shot of Morphine. I just never thought about it really, not until you’re thinking it through, the logistics of it, what that would mean. (0103)

For others, they wished to receive this in their home as this was a familiar environment for their child and where they felt would be most comfortable.

When it does happen, I’d like him to be at home, and have the support at home from the nurses, rather than in hospital or a hospice. I think it’s just so the family, loads of family can come, it’s just nice for them to see him at home and then he’ll be around his things, his bed, his chair, his own environment. I’d like him to be at home. (0616)

However, parents acknowledged even though this was their preference, it might not necessarily be able to be achieved. One parent explained having the option of being able to have end-of-life care in the home, surrounded by family, and able to spend as much time as possible with their child, was really important to them.

At first, it’s going to be as much as possible keep her at home. It’s where she loves, it’s her domain, especially being blind as well, it’s familiarity in her own environment that really helps her but also I think the starkness of hospitals and everything, it’s not what you really want. . . We’d rather prefer to do it at home unless it got to a certain point that it was really unmanageable so that one’s a little bit more changeable. (0510)

COVID-19 restrictions had influenced parents’ choices over preferred place of care. Some had chosen to be at home during their child’s end of life as restrictions on visitors were more flexible, therefore allowing family and friends to be present.

But then [name of child] was really ill. March 2020, the COVID had just started, and we were given the opportunity of going to the local hospice in April, but it meant that I was there with her on my own and family weren’t allowed to go and say goodbye to her or anything. And so that’s why we went home. (0602)

### Parental wishes and unmet needs

This study was informed by Appreciate Inquiry,^14^ Table 5 summarises the Dream Phase, of parental wishes, but also their unmet needs. Inductive analysis of the data, beyond the priori NICE statements, indicated how parents described their unmet needs and indicated their wishes, which they believed would improve palliative care services and support for children (Table 5).

## Discussion

### Main findings

This study explored how the NICE quality standards featured in parental experiences of palliative care for children. Good palliative care is care that is co-led and co-planned with trusted professionals working in palliative care, which is integrated, responsive and flexible, and which encompasses the whole family and enables parents to not only care for, but also to parent their child to end of life.

Findings demonstrated considerable inequalities in access to, and provision of, palliative care, particularly 24/7 care, dependent on age and postcode. Parents often relied on charity funding for resources and support. Parents wanted, but were not able, to focus on being a parent, without additional struggles of fighting for access, budgets, and resources. The importance of introducing palliative care to children and their families in a timely and positive manner, and developing plans for palliative care which focused on quality of life was stressed. Parents highlighted a need to develop better communication between services to ensure continuity, and to better educate professionals who work outside of palliative care, on the meaning and nature of palliative care for children.

### What this study adds

Despite introducing the NICE quality standards in 2017, our study highlights marked differences in parents’ experiences of access to Aoun et al.,^27^ and provision of Mitchell et al.,^28^ and Knapp et al.^29^ palliative and end-of-life care for their children. Parents still have to fight the system to attain palliative care services,^30,31^ particularly 24/7 care. The postcode lottery of receiving palliative care and support is still evident, as is the fragmented nature of services.^30^ These issues were found for all ages, however our study highlights these were more prominent for parents of children living longer with complex healthcare needs. Parents needed to rely on charity funding to bolster support for their children, which did not fulfil gaps in care.^32–34^ This was, and still is, exacerbated by the COVID-19 pandemic.^35^ There is still a pressing issue^30^ to meet the palliative care needs of children and young people. With advancements in medicine and palliative care, children with complex conditions are living longer.^36–38^ Therefore, issues of access and availability of services needs to be rapidly addressed.

Findings stress the importance of introducing palliative care and Advance Care Planning to children and their families in a timely, individualised and positive manner.^28^ Our study shows when plans are co-constructed with a child’s care team, they have the potential to reduce uncertainty for families, conflict with health care professionals, and improve palliative care decision-making for children, which is fraught with emotion, uncertainty and challenge. There is a need to develop better communication between professionals, services and parents to ensure continuity of care, but also a recognition of parental expertise and experience.^28^ Despite NICE guidance, this aspect of care was still highlighted as not working adequately, or translating into practice, with parents advocating for a key person to co-ordinate and drive their child’s care.

Better educating health professionals who were not directly involved in their child’s care, on the meaning, and complexity, of palliative care, is important for ensuring better communication and support.^39^ Findings demonstrate terminology such as ‘palliative care’ is often misunderstood, has negative connotations^9^ and is often associated with imminent death.^30,40,41^ Many parents in our study also highlighted the lack of awareness from professionals working outside of palliative care about other terms such as ‘hospice care’ or ‘respite care’. We suggest this is due to misunderstandings that the trajectories of children with life-limiting conditions are the same as adults.^42^ As an intended enabler to accessing services, outlined in the NICE statements, it would seem understanding of terminology could be viewed as a barrier to accessing good palliative care for children and young people.^43–48^

Differences in experiences of bereavement support were evident, particularly depending of the age of the child and services which families were supported by. Parents of babies reported receiving extensive memory making bereavement support, which they valued.^49^ Families who were engaged with a hospice, stressed the importance of being able to access psychological support during, and after their child’s death, for as long as was needed. Whilst parents whose child had died in hospital, reported memory making activities, strong advocacy was needed to ensure this happened. Whilst this may be partly due to the more intensive, clinical nature of a hospital compared to a hospice environment,^49^ these activities support parents’ engagement with grief tasks and help maintain on-going connections with their child, and are important for parents of children of any age.^50^ Parents also found when they sought help from their GP for bereavement support, they were greeted with hesitancy^51^ as to if and when this should be provided. We posit training in bereavement support for this population is needed for generalist professionals.^51^

Parents viewed their child first and foremost, as a child, rather than defined by their disability or palliative care needs, and wished to be supported to be a parent, particularly at the end of life, and enjoy time with their child. Being able to achieve a family’s preferred place of care, whether this was hospice^49,52^ or home, enabled this. However, for the latter, having access to 24/7 care was necessary in order for this to be an option for families.

### Strengths and limitations of study

A strength of the study was robust analysis, informed by Appreciative Inquiry^14^ which drew out what the features of good palliative care are. The sampling strategy ensured that diverse experiences were included, and therefore information power was attained.^24^ We experienced recruitment challenges due to significant clinical burdens on sites and staff, due to COVID-19. Families indicated whilst they were keen to participate, they had many pressures and demands placed upon them, particularly in relation to healthcare and childcare. Although we achieved a good sample, the voices of parents using hospital palliative care (one site) are not well represented. Over three quarters of the research participants were mothers, making it difficult to differentiate between the experiences of fathers and mothers. Future research should explore the voices of parents using hospital care as well as gender differences in experiences. We also relied on parental report, and there are sometimes differences between parents and children about what matters.

## Conclusion

Despite the introduction of the NICE quality standards there are still considerable variations in parents’ experiences of palliative care for their child, with many standards not translating into practice. This study suggests that good palliative care is care that is co-led and co-planned with trusted professionals working in palliative care; is integrated, responsive and flexible; encompasses the whole family and enables parents to not only care for, but also parent their child to end of life. Good bereavement care starts before a child dies and includes opportunities to make memories for the family as well as providing emotional care spanning a child’s death. These findings have implications for informing evidence-based practice and clinical guidelines.
